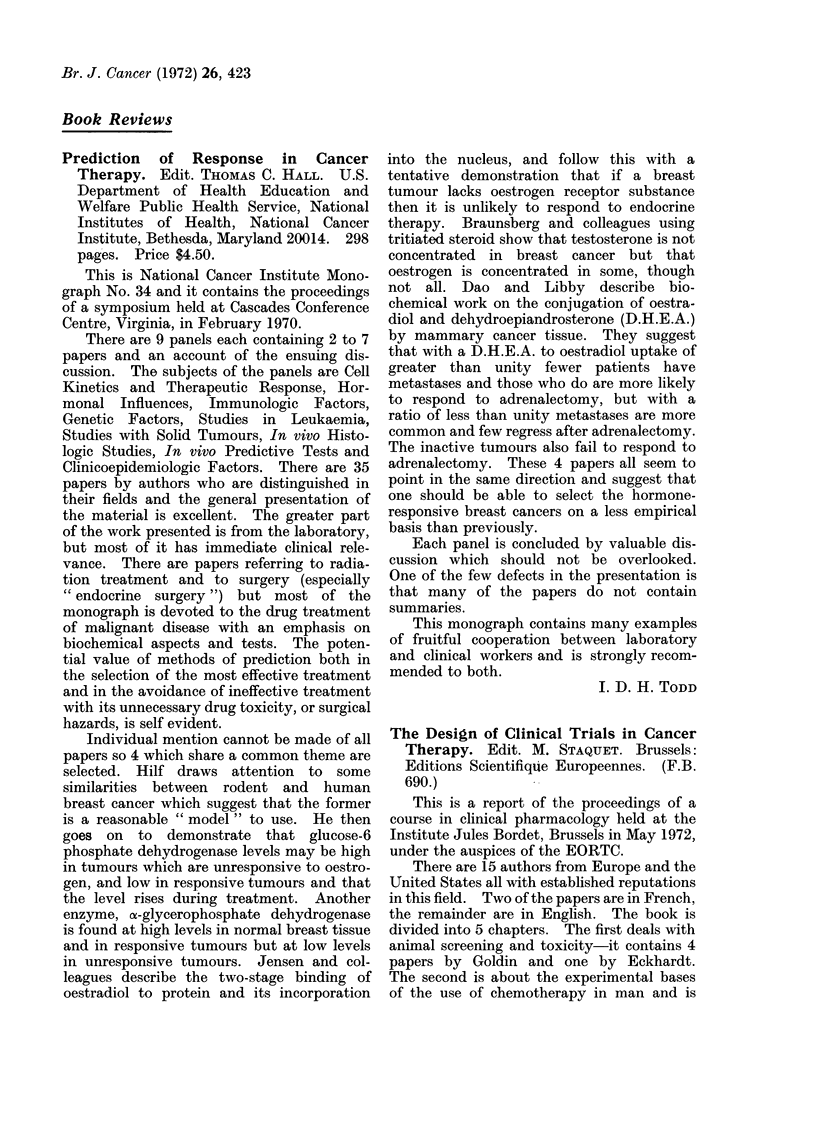# Prediction of Response in Cancer Therapy

**Published:** 1972-10

**Authors:** I. D. H. Todd


*Br. J. Cancer* (1972) **26**, 423

### *Book Reviews*

**Prediction of Response in Cancer Therapy.** Edit. THOMAS C. HALL. U.S. Department of Health Education and Welfare Public Health Service, National Institutes of Health, National Cancer Institute, Bethesda, Maryland 20014. 298 pages. Price $4.50.

This is National Cancer Institute Monograph No. 34 and it contains the proceedings of a symposium held at Cascades Conference Centre, Virginia, in February 1970.

There are 9 panels each containing 2 to 7 papers and an account of the ensuing discussion. The subjects of the panels are Cell Kinetics and Therapeutic Response, Hormonal Influences, Immunologic Factors, Genetic Factors, Studies in Leukaemia, Studies with Solid Tumours, *In vivo* Histologic Studies, *In vivo* Predictive Tests and Clinicoepidemiologic Factors. There are 35 papers by authors who are distinguished in their fields and the general presentation of the material is excellent. The greater part of the work presented is from the laboratory, but most of it has immediate clinical relevance. There are papers referring to radiation treatment and to surgery (especially " endocrine surgery ") but most of the monograph is devoted to the drug treatment of malignant disease with an emphasis on biochemical aspects and tests. The potential value of methods of prediction both in the selection of the most effective treatment and in the avoidance of ineffective treatment with its unnecessary drug toxicity, or surgical hazards, is self evident.

Individual mention cannot be made of all papers so 4 which share a common theme are selected. Hilf draws attention to some similarities between rodent and human breast cancer which suggest that the former is a reasonable " model " to use. He then goes on to demonstrate that glucose-6 phosphate dehydrogenase levels may be high in tumours which are unresponsive to oestrogen, and low in responsive tumours and that the level rises during treatment. Another enzyme, α-glycerophosphate dehydrogenase is found at high levels in normal breast tissue and in responsive tumours but at low levels in unresponsive tumours. Jensen and colleagues describe the two-stage binding of oestradiol to protein and its incorporation into the nucleus, and follow this with a tentative demonstration that if a breast tumour lacks oestrogen receptor substance then it is unlikely to respond to endocrine therapy. Braunsberg and colleagues using tritiated steroid show that testosterone is not concentrated in breast cancer but that oestrogen is concentrated in some, though not all. Dao and Libby describe biochemical work on the conjugation of oestradiol and dehydroepiandrosterone (D.H.E.A.) by mammary cancer tissue. They suggest that with a D.H.E.A. to oestradiol uptake of greater than unity fewer patients have metastases and those who do are more likely to respond to adrenalectomy, but with a ratio of less than unity metastases are more common and few regress after adrenalectomy. The inactive tumours also fail to respond to adrenalectomy. These 4 papers all seem to point in the same direction and suggest that one should be able to select the hormone-responsive breast cancers on a less empirical basis than previously.

Each panel is concluded by valuable discussion which should not be overlooked. One of the few defects in the presentation is that many of the papers do not contain summaries.

This monograph contains many examples of fruitful cooperation between laboratory and clinical workers and is strongly recommended to both.

I. D. H. TODD